# Vincent’s Angina

**Published:** 1912-03

**Authors:** V. M. Griswold

**Affiliations:** Fredonia N. Y.


### Vincent's Angina.

Dr. V. M. GRISWOLD,
Fredonia N. Y.

ULCERO-MEMBRANOUS, or Vincent's angina, is not so rare a disease as published reports might indicate. To quote Burrage (Journ. Am. Med. Association, March 20, 1910), "I have no doubt a positive diagnosis * * * would be made more frequently if, in addition to cultures, smears were made from the pseudo-membrane." The well known difficulty of making cultures of the two symbiotic organisms * * * found in the lesions of this disease is sufficient cause for lack of reports." "Positive diagnosis cannot be made without a microscopic examination of smears from the tonsilar exudates."

Murray (Journ. Am. Med. Association, July 31, 1909) states as follows: "Since Plant, in 1894, described a spirillum, and an associated spindle-shaped bacillus, in cases of membranous tonsilitis, and Vincent in 1896, described similar organisms, in cases of hospital gangrene, a large number of observers have reported similar lesions, in which, the spirillum and bacillus have been constantly associated." "These lesions have been described as acute or subacute, ulcerative, or membranous, inflammations, in which were found the characteristics spirilla and bacilla."

Berkely holds (*Med. News,* May 27, 1905), "that Vincent's angina occurs more frequently than is generally supposed: that it is most frequently confounded with syphilis and diphtheria. He asserts that, when one is familiar with its clinical aspects, a fairly accurate diagnosis may be reached without the microscope."

Mrs. L. F., age 18 years, consulted me September 1, 1910, for pruritis vulva. She was six months along with her first pregnancy.

September 16. The pruritus continued, but was less annoying; but she complained of a slight pharyngitis, for which I prescribed mild treatment.

September 20,. There was on each tonsil a small circular ulcer covered with a whitish, cheesy exudate, easily removed by the local application of persulphate of iron and glycerine. I continued the use of the iron and glycerine alone until the 23d when I added peroxide of hydrogen dilute once in two hours through an atomizer.

---

Read before the Medical Society of Chautauqua Co., N. Y. Dec. 12, 1911.

September 25. Both tonsils still ulcerated and throat painful.

September 26. Patient at work about the house but tonsils ulcerated as before.

October 2. There has been some slight improvement. For several days has had neuralgic pain of face. Began the use of subacetate of copper locally.

October 12. Have used the subacetate of copper daily, and there has been slow, but steady improvement. But the facial pain has persisted. Discovered today that she is cutting her wisdom teeth.

October 15. Have continued the use of the subacetate of copper locally. As the facial pain has persisted sent her to a dentist.

October 17. The facial pain has disappeared.

October 19. The tonsils appear normal. Have continued the use of the subacetate of copper.

October 24. The tonsils are apparently cured. Some pain in the left side of the face. Again sent her to the dentist.

November 6. Delivered her of a seven months foetus, which, manifestly had been dead for some time. She made a somewhat slow recovery.

November 20. Patient discharged.

Resumé. This patient suffered for more than a month with ulcerated tonsils, not at any time alarming, but very resistant to treatment. Here was an anemic young woman, suffering with a complexity of ailments: Pruritus vulvae, haemorrhoids, eruption of wisdom teeth, neuralgic pain of face, and a persistent ulcerative tonsilitis, which, at first, I considered a simple follicular tonsilitis, ordinarily cured by three or four applications of iron and glycerine. But, this treatment failing, I consulted various works on Practice (Tyson, Osler, and others), but failed to find any mention of this condition (I understand, however, that Osler, in his latest edition, mentions Vincent's angina.) But, scattered through various medical journals, I found numerous abstracts and original articles, descriptive of ulcero-membranous, or Vincent's, angina, to which the symptoms in my patient corresponded. I then substituted the subacetate of copper, (a safe and effective application, safe, in that it does not seriously affect the unbroken mucous membrane while its action is readily checked by peroxide of hydrogen), giving at the same time tonic treatment.

The disease is said to be very prevalent, being undetected because of the mildness of the symptoms.

The predisposing causes, to quote Turnicliffe and Turnicliffe, (*N. Y. Med. Jour.* February 17, 1906), "are use of tobacco, trauma to the mucous membrane, as after tonsilotomy, eruption of a wisdom tooth (vide my case) defective teeth or those covered with tartar, alveolar abscesses, gums of scorbutics, syphilis and mercurial stomatitis, acute infectious diseases. No doubt chronic enlargements of the tonsils and adenoids, predispose.

To quote further, from the very exhaustive article by these same authors; "The majority of cases reported have been in young adults between the ages of eighteen and twenty years. But Athanasius' cases were children between the ages of twenty-six months and thirteen years. The cases the former authors observed were children.

Athanasius and others divide the affection into three periods; the onset, characterized by congestion and oedema; the formation of false membrane; and finally the period of ulceration." "The primary location is usually on the tonsils and edge of the gums, but may extend to the tongue, lips, soft palate, pharyngeal wall and cheek. The stomatitis may occur simultaneously, but each is observed alone. With the progress of the disease the ulcer may become much deeper; sometimes leading to destruction of the tonsils, uvula, and parts of the pharyngeal wall. Sometimes the ulceration is very superficial. The submaxillary and retromaxillary glands are usually swollen but they rarely suppurate. The symptoms vary much with the severity of the attack. Often there are no symptoms or only slight ones: The following occur at the onset: Dryness or discomfort of the throat, dysphagia, lassitude, restlessness at night, loss of appetite, headache, coated tongue, constipation, sometimes diarrhoea, vomiting, pains in the abdomen, chills and fever; but usually there is little or no fever. One to five days after the onset the local condition is observed. With it there are pain in swallowing, salivation, and fetid breath, but not always."

Contagion has been observed, but close contact is apparently required, as by means of pipes, pencils, etc.

The prognosis is usually good, but rarely, considerable destruction of tissue may result, and gangrenous processes and noma may develop. Observers have noticed a tendency for the disease to recur after partial or complete recovery."

McWhinney, (*N. Y. Med. Journ.* August 20, 1910) states as follows: "Clinically we must differentiate this lesion from diphtheria and the mucous patch of syphilis." * * * "The aetiologic factor, is the presence of Vincent's spirillium and the fusiform bacillus."

Plant, in 1894, first described a spirillum and spindle-shaped bacillus in cases of membranous amygdalitis. Vincent, in 1896, described similar organisms in cases of hospital gangrene. * * * "The bacillus varies from ten to twelve micra in length, occasionally as short as six micra, and is usually, though not always, associated, with a fine spirillum, extremely long, and on a hanging drop slide, is seen as motile. It is a saprophyte of the buccal cavity but becomes pathogenic in the presence of other organisms. While any of the aniline dyes may be used, the intensity is greatest when gentian violet or aqueous fuchsin is used." Personally, he states, he can recall three cases that were diagnosed as diphtheria, and four as syphilis, clinically, which microscopically revealed a characteristic fusiform bacillus and spirillum. He also states that other organisms are found; often the streptococcus; but the exudate is as a rule characteristic, usually grayish or whitish, and often with a pronounced adenitis." "Cases in which the membrane is thin rarely give rise to much depression. This is not present to a marked degree except where the membrane covers a large area of the tonsil or pharynx, and is of the heavier type."

He reports two cases:

Mr. R. A. N., age 45 years, four month ago had a severe attack of amygdalitis, and a short time after both tonsils were covered with a grayish membrane. * * * Other than difficult deglutition and some depression he felt well. The tonsils were amputated three times at intervals of a month; the membrane recurring. When I examined the case I found on the tonsil stumps a membranous formation; temp. 99.2; pulse 74. Microscopically, by means of smears, Vincent's spirillum and fusiform bacillus were demonstrated. The stumps were enucleated, the capsules completely taken out by the finger, fossae and surrounding area treated for three days with tincture of iodin full strength. Time elapsed since, four months. From the preceding history, and elapsed time, I believe I have effected a cure."

Case 2. Mrs. J. H., age 28 years, sore throat on right side four days. Temperature 99 to 103 but with a pulse of only 86. Had been treated one week as amygdalitis. The right tonsil was found to be swollen and a distinct ulceration seen, two thirds of which, was covered with a grayish or slate colored exudate. Incisions had been previously made above for possible peritonsilar abscess, and these wounds had at once been covered by this exudate. There was great depression, but at the end of four days treatment she was able to eat and on the sixth day went out."

Filatoo and Nevegen, consider chlorate of potassium internally a specific. Crandall, also found that chlorate of potassium did most good. Tincture of iodin is considered effective. Because of the anaerobic character of the bacillus, peroxide of hydrogen is most useful, applied directly to the seat of the disease.

Sobel and Herrman, used 3 per cent. to 5 per cent. solution of silver nitrate applied daily.

Murray, used peroxide of hydrogen, followed by silver nitrate, 2 per cent. to 10 per cent. and 5 per cent. zinc sulphocarbolate. In three weeks the pseudo-membrane disappeared, and the throat presented a normal appearance.

Fraly, used 50 per cent. argyrol solution after liquor antisepticus, and peroxide of hydrogen, every three hours.

Methylene blue has been used locally. Also iodide of potassium internally.

E. H. Place (*Boston Med. and Surg. Journ.,* November 9, 1911), states, that in his hands, the most satisfactory of the local applications, and one universally successful in a few days, has been swabbing with peroxide of hydrogen full strength or diluted one half, until the ulcer is pretty clean, and then painting with a 2 per cent. solution of chromic acid once daily; the combination working much better than either alone. Cure occurring rapidly in two to six days with hardly an exception. Removal of the tartar from the teeth and oral hygiene are important. He believes that the disease is preventable by proper attention to the teeth, mouth, and general condition. "Vincent's angina should become as unknown as hospital gangrene or typhus fever."

On the whole, the most effective treatment seems to be, tinct. of iodin locally once a day, after thorough swabbing with peroxide of hydrogen, or with the solution of chromic acid as used by Place; with chlorate of potassium internally. However, my patient recovered under the use of subacetate of copper locally, followed by peroxide of hydrogen to neutralize the copper; peroxide spraying, and tonic treatment.

51, East Main St.

---

Nasal Diphtheria, N. A. Christie, M. B., Webwood, Ont., in *Can. Pract. & Review,* January 1912. Male, aged 17, membrane on tonsils and uvula and visible through anterior nares. 4,000 units July 30, 10,000, July 31st. Much cellulitis pushing lower jaw forward. August 5, hæmorrhage from right nostril. Almost complete cast, ⅛ inch thick, expelled on blowing nose. Similar cast expelled during following night. Hæmorrhage controlled by Tr. ferri chlor. Recovery complicated with diplopia, pharyngeal paralysis, anaemia and general muscular weakness.

